# Supplementary material for: An incoherent feedforward loop interprets NFκB/RelA dynamics to determine TNF‐induced necroptosis decisions
Source: Mol Syst Biol. 2020 Dec 14;16(12):e9677. doi: 10.15252/msb.20209677 (PMC7734648; doi:10.15252/msb.20209677)
Supplement: Supplementary file 1 — Appendix [file MSB-16-e9677-s001.pdf]

APPENDIX

Contents

Cell-death conceptual model 2
Model construction 2
Model simulations 2
Death time distribution 2
Parameter scan 2
Mathematic model for TNF-induced necroptosis 4
Model construction 4
TNFR-NFκB-4-IκB module 4
Necroptosis module 4
Model simulations 4
Wildtype 4
RelA-knockout 4
A20-knockout 4
Constitutive A20 expression 5
TNF pulse stimulation of different durations 5
Synthetically regulated NFκB 5
Appendix Tables for cell-death conceptual models 6
Appendix Table S1: Model species for cell death conceptual model 6
Appendix Table S2: Parameters for cell death conceptual models 6
Appendix Table S3: Distributed parameters for cell death conceptual model 6
Appendix Table S4: Perturbed range of parameters for cell death conceptual model 6
Appendix Tables for TNF-induced necroptosis model 7
Appendix Table S5: Model species 7
Appendix Table S6: Model reactions 7
Appendix Table S7: Perturbed parameters for NFκB module 10
Appendix Table S8: Parameters for necroptosis module 10
Appendix Table S9: Distributed parameters for necroptosis model 10
References 11

Cell-death conceptual model

Model construction

To explore whether differential expression dynamics of a constitutive or stimulus-induced survival factor would produce differential time courses in the incidences of cell death, we constructed two conceptual models representing core network motifs of TNF-induced necroptosis as depicted in Figure 1. In the first, TNF induced activation of RIPK1/3 and the necroptosis effector pMLKL is counteracted by an unknown, constitutively expressed survival factor X. In the second model, TNF also induces IκB-controlled NFκB, which in turn induces the expression of the survival factor X. The modeling species and their initial values are listed in Appendix Table 1.

IκB-controlled NFκB activity is a coarse-grained version of previously published models (e.g. Werner et al. 2008), which we extended by adding inducible expression of survival factor X. The dynamics of each species were modeled by ordinary differential equations with synthesis and degradation terms (Kærn et al. 2003, Bintu et al. 2005, Krishna et al. 2006, Alon 2007, Ma et al., 2009, Tyson et al. 2010, Tang et al. 2017). The synthesis term contains the activation and inhibition as a product of Hill functions (MacArthur et al., 2009). The Hill functions have a sigmoidal shape suitable to represent the cascade-like activation of the protein kinases RIPK1/3 and pMLKL, which behave like a highly cooperative enzymatic reaction (Huang et al. 1996, Kapuy et al. 2009, Shu et al. 2013). The equations are stated below:

$$
\begin{aligned}
TNF &= \exp(-t/600),\\
\frac{d[RIP1]}{dt} &= k_{s1}\frac{(TNF/Km_1)^{n_1}}{(TNF/Km_1)^{n_1}+1}\frac{1}{([X]/Km_5)^{n_5}+1} - k_{d1}[RIP1],\\
\frac{d[pMLKL]}{dt} &= k_{s2}\frac{([RIP1]/Km_2)^{n_2}}{([RIP1]/Km_2)^{n_2}+1} - k_{d2}[pMLKL],\\
\frac{d[X]}{dt} &= -k_{d3}[X]/3,\\
\frac{d[IkB]}{dt} &= 0,\\
\frac{d[NFkB]}{dt} &= 0.
\end{aligned}
$$

$$
\begin{aligned}
TNF &= \exp(-t/600),\\
\frac{d[RIP1]}{dt} &= k_{s1}\frac{(TNF/Km_1)^{n_1}}{(TNF/Km_1)^{n_1}+1}\frac{1}{([X]/Km_5)^{n_5}+1} - k_{d1}[RIP1],\\
\frac{d[pMLKL]}{dt} &= k_{s2}\frac{([RIP1]/Km_2)^{n_2}}{([RIP1]/Km_2)^{n_2}+1} - k_{d2}[pMLKL],\\
\frac{d[X]}{dt} &= k_{s3}\frac{([NFkB]/Km_3)^{n_3}}{([NFkB]/Km_3)^{n_3}+1} - k_{d3}[X],\\
\frac{d[IkB]}{dt} &= k_{s4}\frac{([NFkB]/Km_4)^{n_4}}{([NFkB]/Km_4)^{n_4}+1}\frac{1}{(TNF/Km_6)^{n_6}+1} - k_{d4}[IkB],\\
\frac{d[NFkB]}{dt} &= k_{s5}\frac{1}{([IkB]/Km_7)^{n_7}+1} - k_{d5}[NFkB].
\end{aligned}
$$

The parameters include synthesis rates k_s, degradation rates k_d, Hill coefficients n, and Km values in the Hill functions. The synthesis and degradation rates determine the activation and decay rate of the curves of protein concentration; Hill coefficients correspond to the sensitivity upon activation; and Km values denote the dependence of activation on the concentration of activator or inhibitor. We assumed that all the parameters are constant, as listed in Appendix Table 2. The unit of time in the simulation is minute. To account for cell-to-cell heterogeneity in expression of survival factor X (Friedman et al. 2006, Shalek et al. 2013), we distributed either its constitutive expression in the first model, or the NFκB-responsive synthesis rate in the second model. The distributed parameters are listed in Appendix Table 3.

Model simulations

Death time distribution

For each model, we simulated 300 single cells, with each simulation sampling one set of values from the parameter distributions. Previous evidence suggests that a threshold mechanism exists for pMLKL, which must be crossed in order to effectively induce necroptosis (Gong et al. 2017, Samson et al. 2020). Thus, we model the irreversible cell death event with pMLKL reaching a threshold (Spencer et al. 2009, Roux et al. 2015), and the death time is registered as the time of threshold crossing. The threshold is a relative value to the rate of pMLKL accumulation, and kept constant between both versions of the model to compare relative differences in necroptosis kinetics caused by constitutive and stimulus-induced inhibitors. The conceptual modeling framework can be dimensionless, and we added units that are consistent with the more extensive model of necroptosis below. Since only the relative value of protein to the threshold determines the cell death time, the protein values were normalized within a certain range with arbitrary units (A.U.). We conducted these simulations using MATLAB® file DeathConcept2.m. All simulations were done using MATLAB® version R2019a.

In Figure 1, we plotted the time course of cells undergoing death by hourly binning the number of simulations in which pMLKL exceeds the threshold. While the first model returns a unimodal, the second model returns a non-unimodal distribution of death times.

Parameter scan

These two conceptual models aim to provide a coarse-grained description of necroptosis kinetics. As we aimed to compare relative differences in necroptosis kinetics between both versions depending on whether constitutive or stimulus-induced survival factors are present, the absolute parameter values were not critical. However, the dynamics of IκB-controlled NFκB were adjusted to coarsely match previously published models (Werner et al. 2008, Krishna et al. 2006). To implement dependence of pMLKL on the stimulus and protective protein, we further adjusted the dynamics of upstream proteins. For example, we chose Km values in the range of the concentration of the activators or inhibitors during their transient dynamics, such that the activation or inhibition took effect when the protein accumulates to the Km values. The synthesis and degradation rates of upstream proteins were set accordingly to ensure the proteins reached Km values to affect the downstream proteins during the time window of simulation. We allowed the effective Hill coefficients in the range of [1,5] (Goldbeter et al. 1981, Huang et al. 1996). The Hill coefficient was adjusted to tune the sensitivity of the activation or inhibition: if downstream protein was sharply affected by the activator or inhibitor, the relatively large Hill coefficient was implied.

In order to test the dependence of cell death time distributions on the parameters, we performed parameter sensitivity analysis for the synthesis rates, degradation rates and Km values of all reactions (Appendix Figure 1). For each simulation, we sampled one set of parameters from the parameter regime of 2-fold higher and 2-fold lower relative to the original values above, as in Appendix Table 4. We then performed statistical analysis of the distributions of death times to calculate a probability of unimodal distributions as explained in the Methods section (Hartigan et al. 1985). We classified the cell death time distribution as unimodal or non-unimodal by separating the unimodality score at the threshold 0.5.

We repeated the simulation 1000 times for each model, and classified the returned death time distributions into 3 types: (1) "survival ratio > 40%" with overall survival of > 40% of the cell population, (2) "unimodal" with overall survival of < 40% and a unimodality score of > 0.5, (3)

"non-unimodal" with overall survival of < 40% and a unimodality score of < 0.5. We excluded simulations with > 40% survival to increase the confidence in classifying the modality of death time distribution (Appendix Figure 1). The simulations showed that the first model majorly returns unimodal distributions, whereas the second model preferably generates non-unimodal distributions of death times (Appendix Figure 1).

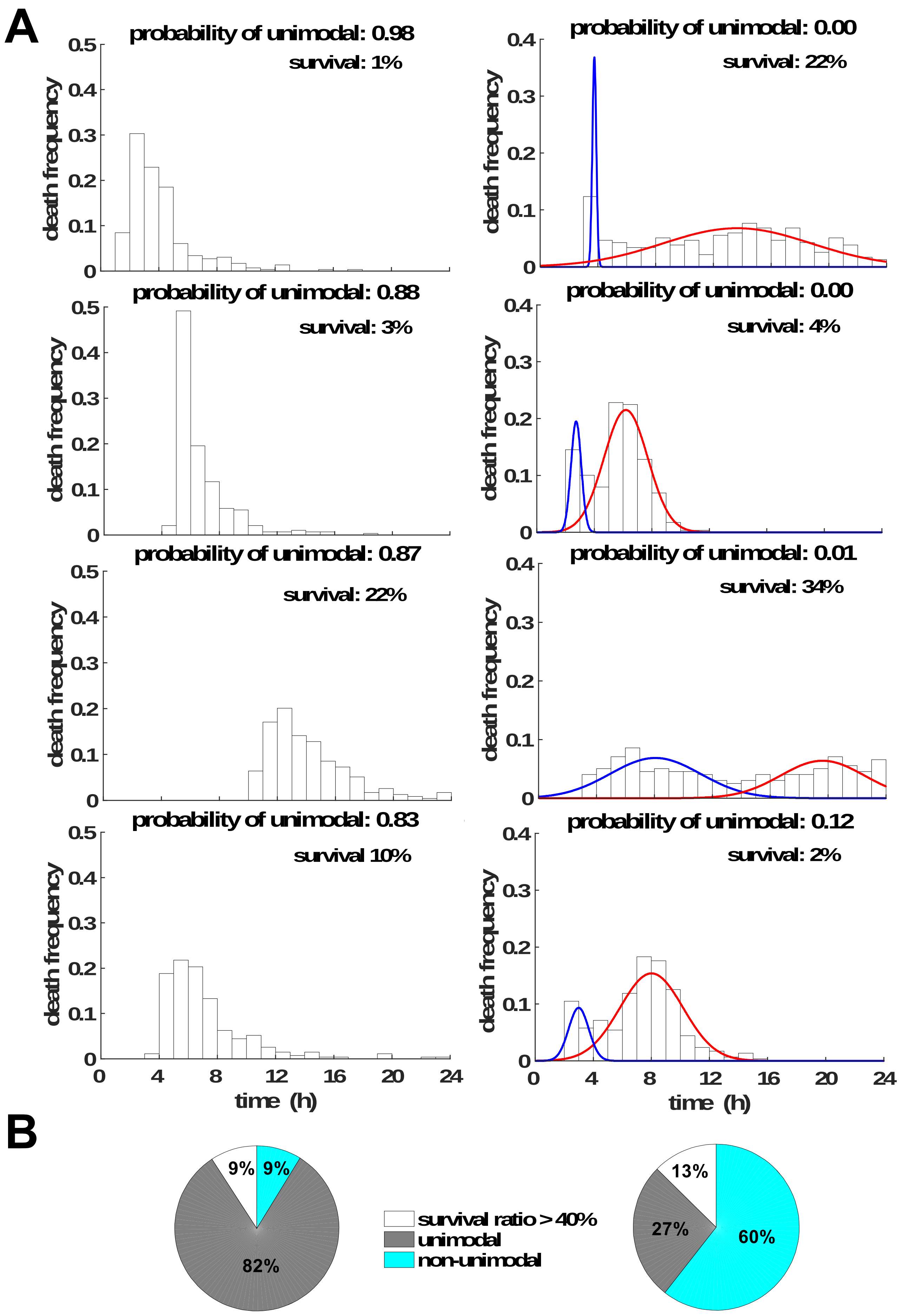

Appendix Figure S1: Parameter sensitivity analysis reveals non-unimodal death time distributions as a robust feature of inducible survival mechanisms.
(A) Representative simulations of death times after parameter sensitivity analysis for both conceptual modeling versions of TNF-induced necroptosis (left: constitutive, right: inducible survival factor). Probability of unimodality calculated by Hartigan's dip significance test, and classified as unimodal or non-unimodal at threshold 0.5. (B) Percentages of simulations revealing unimodal or non-unimodal distributions of death times; simulations with >40% overall survival were excluded.

Mathematic model for TNF-induced necroptosis

Model construction

To construct a mechanistic model of NFκB and necroptosis network dynamics, we first combined the previously published model of TNFR signaling to NFκB (Werner et al. 2008) with the 4-IκB-containing NFκB module (Shih et al. 2009). This merged version reflects TNF-induced activation of complex I to activate IKK, which causes the degradation of IκB proteins and activation of NFκB to initiate transcription of IκBs as well as A20, with A20 counteracting the activation of complex I (Figure EV3A). In a next step, we extended this model by adding a course-grained necroptosis module depicting complex I activating RIPK1, RIPK3 and the effector pMLKL, with A20 inhibiting the activation of RIPK3 (Figure EV3A). While this model of necroptosis is limited to the core biochemical signaling processes, it was sufficient to recapitulate TNF-induced signaling kinetics controlled by NFκB activity dynamics.

TNFR-NFκB-4-IκB module

We adapted the module of TNF signaling to NFκB via IKK from [Werner et al. 2008 G&D], which includes the negative feedback regulators IκBα, IκBε, and IκBβ as well as the regulator A20. For completeness, we have listed all species, reactions, initial values and the parameters in Appendix Table 5 and Appendix Table 6. We then added the 4-IκB-containing NFκB module from (Shih et al. 2009) to include IκBδ inhibitory activity. We listed all species, reactions, initial values and parameters in Appendix Table 5 and Appendix Table 6 as well. To parameterize this model to L929 cells, we used experimental measurements of NFκB activity (EMSA) and IκB expression (Immunoblot). TNF half-life was set to 10 hours to match our data. All changes are listed in Appendix Table 7.

Necroptosis module

To quantitatively understand the kinetics of necroptosis signaling, we constructed a minimal model to describe the dynamics of the three molecular species RIPK1, RIPK3, and pMLKL (active forms of all regulators). Each equation is composed of synthesis and decay terms (Kærn et al. 2003, Bintu et al. 2005, Alon 2007, Shu et al. 2013, Ma et al., 2009, Tyson et al. 2010, Tang et al. 2017). Similar as the model in Fig.1, the synthesis term is a product of activation and inhibition by other signaling proteins following the schematic, and modeled by a product of Hill functions (MacArthur et al. 2009). The inhibition of necroptosis by A20 is also modeled by a Hill function in the synthesis term from RIP1 to RIP3. The equations are stated below:

$$\frac{d[RIP1]}{dt} = k_{s1}\frac{[([C1]+[C1_off])/Km_1]^{n_1}}{[([C1]+[C1_off])/Km_1]^{n_1}+1} - k_{d1}[RIP1],$$
$$\frac{d[RIP3]}{dt} = k_{s2}\frac{([RIP1]/Km_2)^{n_2}}{([RIP1]/Km_3)^{n_2}+1}\frac{1}{([A20]/Km_3)^{n_3}+1} - k_{d2}[RIP3],$$
$$\frac{d[pMLKL]}{dt} = k_{s3}\frac{([RIP3]/Km_4)^{n_4}}{([RIP3]/Km_4)^{n_4}+1} - k_{d3}[pMLKL].$$

The parameters contain synthesis rates, degradation rates, Hill coefficients, and Km values. They were fit to experimental measurements of biochemical markers of necroptosis (pMLKL and RIPK3 measurements via Western blot), death kinetics (microscopy) and fractional survival (microscopy and/or crystal violet assay). For example, the synthesis and degradation rates were determined by the activation rate of the time course data of RIPK1, RIPK3 and pMLKL; Hill coefficients were adjusted to fit the steepness of the activation curve upon stimulation; and Km values were chosen to match the dependence on the concentration of activator or inhibitor. We repeated this fitting procedure until the simulated time course of signaling proteins is fitted with their measured activity at different time points, with the parameters in Appendix Table 8.

Model simulations

Wildtype

We simulated 300 cells comparable to each microscopy experiment, and simulated the time course of each molecular species in the model. To account for cell-to-cell heterogeneity, we distributed a minimal number of parameters: RIPK1 to RIPK3 activation rate, which obeys a unimodal distribution (equivalent to unknown constitutive survival mechanisms); and A20 mRNA synthesis rate, which follows a bimodal distribution in line with our single-molecule FISH measurements. All the distributed parameters are listed in Appendix Table 9. We used the population average of simulated mRNA and/or protein concentration to fit with experimental measurements for NFκB, IκB, A20, RIPK3 and pMLKL. We plotted the simulated time course along with experimental measurements for key molecules, such as nuclear NFκB, A20 mRNA, A20 protein, RIP3, and pMLKL (Figure EV3B-G). The shaded area is the range of the 20th percentile around the median value of the simulated 300 cells. For each species, we first normalized the data to simulation, since the simulation has the protein in the unit of μM and time in the unit of minute. After fitting to the data, we renormalized both the data and simulation back to value of experiment, such that the figures are in the same scale of original normalized data under an arbitrary unit (A.U.).

To track death events, we used a threshold for pMLKL similarly to the conceptual model. Having verified (i) a linear relationship between pMLKL concentration and detection in experimental immunoblot studies (Figure EV1E), and (ii) approximate correlation between these measurements and necroptosis (microscopy assay) in a time course of TNF treatment (Figure 1I), we described pMLKL concentration by an arbitrary unit relative to these experimental measurements (Figure EV3E), and subsequently parameterized the rate of pMLKL accumulation and the threshold to generate similar fractional survival in the same time scale, i.e. 24 hours of TNF treatment. We then plotted the death time distributions, death rates and fractional survival (Figure 3B, C). Using the distributed parameters, the repeated simulations of single cells recapitulated the bimodal death time distributions, two-phased death rates and fractional survival of experimental measurements. The simulations were executed in the MATLAB® script NecroptosisMain1.m.

RelA-knockout

After validating that the model accurately recapitulated the time course of key signaling molecules, as well as the death time distribution of wildtype cells, we applied it to simulate the scenario of NFκB/RelA-knockout cells (RelA KO). We modeled RelA KO by setting the value of NFκB (RelA) to zero. Without RelA, TNF did not induce A20 expression, and therefore cell death occurred more rapidly within the early phase of the simulated time course. Accordingly, the simulation returned unimodal death time distributions, single-phased death rates and matched overall fractional survival of experimental measurements. The simulated time course is plotted in Figure 3B, C. We concluded that the model can accurately recapitulate the RelA-dependent regulation of necroptosis kinetics.

A20-knockout

Similarly, we modeled the scenario of A20-knockout (A20 KO) by setting A20 mRNA to zero. Without the protection of A20 protein, cell death occurred rapidly within the early phase of the simulated time course, and followed a unimodal shape, which was in line with measurements (Figure EV3I). It demonstrated that our model properly fits the strength of A20 inhibition on cell death.

Constitutive A20 expression

Next, we explored whether constitutive or transient A20 expression is crucial for the regulation of necroptosis kinetics. After setting TNF-inducible expression of A20 to zero, we simulated 24 hours of TNF treatment with constitutive A20 transcription rates modified by factors of 2^x (x = -1, 0, 1; "basal / RelA-knockout" resembled by x = -1). We simulated 300 cells per condition and plotted the resulting death time distributions as death count over time (Figure 3D).

TNF pulse stimulation of different durations

We applied the model to simulate overall fractional survival after different duration doses of TNF (10 ng/ml). By increasing the decay rate of TNF (1500-fold) at 3, 6, 12, or 24 hours, TNF soon reaches zero after those time points, respectively. As TNF duration increased, the simulated fractional survival of wildtype or RelA-knockout cells at 24 hours decreased (Figure 3I). The model predictions were tested experimentally by washing out TNF at chosen time points (Figure 3J).

Synthetically regulated NFκB

In order to explore how the duration of A20 expression controls overall fractional survival in response to TNF, we placed the A20 promoter under the control of a "synthetic" NFκB activity. Synthetically regulated NFκB activity was implemented using a pulse function, which is not restrained by IκB feedback regulators and serves as an input to control mA20 expression. The pulse function of NFκB activity started at time zero with 3 nM, and increased to be 83 nM, and we tested a set of durations: 0.5, 1, 2, 4, 8, 16 hours. Correspondingly, the simulated induction of mA20 followed similar dynamics, as shown in Figure 4A, B. As the duration of synthetic NFκB activity and subsequent mA20 expression increased, the fractional survival at 24 hours increased as well. We validated this prediction experimentally by using IκBα/IκBε-knockout cells showing elevated NFκB activity and prolonged expression of mA20 (Figure 4C-G). These findings suggested that dysregulated activation of NFκB and inducible A20 may protect cells from cytotoxic functions of long-term TNF-exposure.

Appendix Tables for cell-death conceptual models

Appendix Table 1: Model species for cell death conceptual model

	Model Species	Initial value in Model 1	Initial value in Model 2
**1**	RIP1/3	0	0
**2**	pMLKL	0	0
**3**	X	0.05*lognormal(0,0.3) μM, where lognormal(a,b) is a log-normal distribution with mean a and variable b.	0
**4**	IκB	0	0
**5**	NF-κB	0	0.01 μM

Appendix Table 2: Parameters for cell death conceptual models

#	Reaction	Parameter	Parameter Value	Model 1	Model 2
	TNF decay	Half life	600 min	Yes	Yes
1	TNF --> RIP1/3, X --| RIP1/3	Synthesis rate k_{s1}	0.1 min^{-1}	Yes	Yes
2	RIP1/3 --> pMLKL	Synthesis rate k_{s2}	0.1 min^{-1}	Yes	Yes
3	NFkB --> X	Synthesis rate k_{s3}	5 min^{-1}	No	Yes
4	TNF --| IkB, NFkB --> IkB	Synthesis rate k_{s4}	5 min^{-1}	No	Yes
5	IkB --| NFkB	Synthesis rate k_{s5}	1 min^{-1}	No	Yes
1	RIP1/3 =>	Degradation rate k_{d1}	0.01 μM^{-1} min^{-1}	Yes	Yes
2	pMLKL =>	Degradation rate k_{d2}	0.1 μM^{-1} min^{-1}	Yes	Yes
3	X =>	Degradation rate k_{d3}	1 μM^{-} min^{-1}	No	Yes
4	IkB =>	Degradation rate k_{d4}	1 μM^{-1} min^{-1}	No	Yes
5	NFkB =>	Degradation rate k_{d5}	1 μM^{-1} min^{-1}	No	Yes
1	TNF --> RIP1/3	Km_1	1 μM	Yes	Yes
2	RIP1/3 --> pMLKL	Km_2	0.15 μM	Yes	Yes
3	NFkB --> X	Km_3	0.5 μM	No	Yes
4	NFkB --> IkB	Km_4	0.3 μM	No	Yes
1	X --| RIP1/3	Km_5	0.3 μM	Yes	Yes
4	TNF --| IkB	Km_6	0.3 μM	No	Yes
5	IkB --| NFkB	Km_7	0.03 μM	No	Yes
1	TNF --> RIP1/3	Hill coefficient n_1	3	Yes	Yes
2	RIP1/3 --> pMLKL	Hill coefficient n_2	3	Yes	Yes
3	NFkB --> X	Hill coefficient n_3	3	No	Yes
4	NFkB --> IkB	Hill coefficient n_4	3	No	Yes
1	X --| RIP1/3	Hill coefficient n_5	3	Yes	Yes
4	TNF --| IkB	Hill coefficient n_6	3	No	Yes
5	IkB --| NFkB	Hill coefficient n_7	3	No	Yes

Appendix Table 3: Distributed parameters for cell death conceptual model

#	Parameter	Parameter Value	Parameter distribution
3	Synthesis rate k_{s3}	Distributed	0.5*normal(7,8)+ 0.5*normal(14,2), where normal(a,b) is a normal distribution with mean a and variable b.

Appendix Table 4: Perturbed range of parameters for cell death conceptual model

#	Parameter	Parameter Value	Sampling range
1	Synthesis rate k_{s1}	0.1 min^{-1}	[0.5, 1, 2]
2	Synthesis rate k_{s2}	0.1 min^{-1}	[0.5, 1, 2]
3	Synthesis rate k_{s3}	5 min^{-1}	[0.5, 1, 2]
4	Synthesis rate k_{s4}	5 min^{-1}	[0.5, 1, 2]
5	Synthesis rate k_{s5}	1 min^{-1}	[0.5, 1, 2]
1	Degradation rate k_{d1}	0.01 μM^{-1} min^{-1}	[0.5, 1, 2]
2	Degradation rate k_{d2}	0.1 μM^{-1} min^{-1}	[0.5, 1, 2]
3	Degradation rate k_{d3}	1 μM^{-1} min^{-1}	[0.5, 1, 2]
4	Degradation rate k_{d4}	1 μM^{-1} min^{-1}	[0.5, 1, 2]
5	Degradation rate k_{d5}	1 μM^{-1} min^{-1}	[0.5, 1, 2]
1	Km_1	1 μM	[0.5, 1, 2]
2	Km_2	0.15 μM	[0.5, 1, 2]
3	Km_3	0.5 μM	[0.5, 1, 2]
4	Km_4	0.3 μM	[0.5, 1, 2]
1	Km_5	0.3 μM	[0.5, 1, 2]
4	Km_6	0.3 μM	[0.5, 1, 2]
5	Km_7	0.03 μM	[0.5, 1, 2]

Appendix Tables for TNF-induced necroptosis model

Appendix Table 5: Model species

	Model Species	Model Nomenclature	Initial μM	Location
**1**	IκBα	IkBa	0	Cytoplasm
**2**	IκBα	IkBan	0	Nucleus
**3**	IκBα-NFκB	IkBaNFkB	0	Cytoplasm
**4**	IκBα-NFκB	IkBaNFkBn	0	Nucleus
**5**	IκBα mRNA	IkBat	0	Cytoplasm
**6**	IκBβ	IkBb	0	Cytoplasm
**7**	IκBβ	IkBbn	0	Nucleus
**8**	IκBβ-NFκB	IkBbNFkB	0	Cytoplasm
**9**	IκBβ-NFκB	IkBbNFkBn	0	Nucleus
**10**	IκBϵ mRNA	IkBbt	0	Cytoplasm
**11**	IκBϵ	IkBe	0	Cytoplasm
**12**	IκBϵ	IkBen	0	Nucleus
**13**	IκBϵ-NFκB	IkBeNFkB	0	Cytoplasm
**14**	IκBϵ-NFκB	IkBeNFkBn	0	Nucleus
**15**	IκBϵ mRNA	IkBet	0	Cytoplasm
**16**	IκBδ	IkBd	0	Cytoplasm
**17**	IκBδ	IkBdn	0	Nucleus
**18**	IκBδ-NFκB	IkBdNFkB	0	Cytoplasm
**19**	IκBδ-NFκB	IkBdNFkBn	0	Nucleus
**20**	IκBδ mRNA	IkBdt	0	Cytoplasm
**21**	NF-κB	NFkB	0	Cytoplasm
**22**	NF-κB	NFkBn	0.125	Nucleus
**23**	TAK1 (inactive)	IKKK_off	0.1	Cytoplasm
**24**	TAK1 (active)	IKKK	0	Cytoplasm
**25**	IKK (inactive)	IKK_off	0.1	Cytoplasm
**26**	IKK (active)	IKK	0	Cytoplasm
**27**	IKK (auto-inactivated)	IKK_i	0	Cytoplasm
**28**	TNF	tnf	0	Extracellular
**29**	TNF Receptor Monomer	tnfrm	0	Cell Surface
**30**	TNF Receptor Trimer	TNFR	0	Cell Surface
**31**	TNF-Bound TNF Receptor Trimer	TNFRtnf	0	Cell Surface
**32**	TNFR Complex I (active)	C1	0	Cell Surface
**33**	TNFR Complex I (inactive)	C1_off	0	Cell Surface
**34**	TNF-Bound TNFR Complex I (active)	C1tnf	0	Cell Surface
**35**	TNF-Bound TNFR Complex I (inactive)	C1_tnf_off	0	Cell Surface
**36**	TRAF-TRADD-RIP	TTR	8.3e-4	Cytoplasm
**37**	A20	A20	0	Cytoplasm
**38**	A20 mRNA	A20t	0	Cytoplasm
**39**	RIP3	RIP3	0	Cytoplasm
**40**	pMLKL	pMLKL	0	Cytoplasm
**41**	RIP1	RIP1	0	Cytoplasm

Note for Appendix Table 5: There are 41 species in the model. Each is represented by a unique name (nomenclature), with an initial concentration and cellular localization.

Appendix Table 6: Model reactions

**NFκB Activation Module**					
IκB mRNA and Protein Synthesis Reactions					
**#**	**Reaction**	**Parameter Value**	**Category**	**Location**	**Source of Parameter Value**
**1**	=> IkBat (constitutive)	7 E-5 min^{-1}	RNA Synth.	-	Parameter value chosen to fit mRNA and protein expression profiles as measured by RNase Protection (RPA) and Western Blot assays.
**2**	=> IkBbt (constitutive)	1 E-5 min^{-1}	RNA Synth.	-	Refer to #1.
**3**	=> IkBet (constitutive)	1 E-6 min^{-1}	RNA Synth.	-	Refer to #1.
**72**	=> IkBdt (constitutive)	1 E-7 min-1	RNA Synth.	-	Refer to #1.
**4**<br>**7**<br>**10**	=> IkBat (induced by NFkBn)	8 μM^{-2} min^{-1}<br>Hill Coefficient: 3.0<br>Delay: 0 min	RNA Synth.	-	(Werner et al., 2005)<br>(Werner et al., 2005)<br>(Kearns et al., 2006) and unpublished results
**5**<br>**8**<br>**11**	=> IkBbt (induced by NFkBn)	0.02 μM^{-2} min^{-1}<br>Hill Coefficient: 3.0<br>Delay: 37 min	RNA Synth.	-	(Kearns et al., 2006)<br>(Werner et al., 2005)<br>(Kearns et al., 2006) and unpublished results
**6**<br>**9**<br>**12**	=> IkBet (induced by NFkBn)	0.3 μM^{-2} min^{-1}<br>Hill Coefficient: 3.0<br>Delay: 37 min	RNA Synth.	-	(Kearns et al., 2006)<br>(Werner et al., 2005)<br>(Kearns et al., 2006) and unpublished results
**73**<br>**74**<br>**75**	=> IkBdt (induced by NFkBn)	0.025 μM^{-2} min^{-1}<br>Hill Coefficient: 3.0<br>Delay: 90 min	RNA Synth.	-	(Shih et al., 2009)
**13**	IkBat =>	0.035 min^{-1}	RNA Deg.	Cytoplasm	mRNA half-life measurements using actinomycin-D treatment of cells and RPA. (unpublished results)
**14**	IkBbt =>	3 E-3 min^{-1}	RNA Deg.	Cytoplasm	Refer to #7.
**15**	IkBet =>	4 E-3 min^{-1}	RNA Deg.	Cytoplasm	Refer to #7.
**76**	IkBdt =>	2 E-3 min^{-1}	RNA Deg.	Cytoplasm	Refer to #7.
**16**	=> IkBa	0.25 min^{-1}	Prot. Synth.	Cytoplasm	(Hoffmann et al., 2002)

**17**	=> IkBb	0.25 min^{-1}	Prot. Synth.	Cytoplasm	(Hoffmann et al., 2002)
**18**	=> IkBe	0.25 min^{-1}	Prot. Synth.	Cytoplasm	(Hoffmann et al., 2002)
**77**	=> IkBd	0.25 min^{-1}	Prot. Synth.	Cytoplasm	(Shih et al., 2009)
IκB and NFκB Cellular Localization Reactions					
**19**	IkBa => IkBan	0.09 min^{-1}	Import	-	(Werner et al., 2005)
**20**	IkBb => IkBbn	0.009 min^{-1}	Import	-	(Werner et al., 2005)
**21**	IkBe => IkBen	0.045 min^{-1}	Import	-	(Werner et al., 2005)
**78**	IkBd => IkBdn	0.045 min^{-1}	Import	-	(Shih et al., 2009)
**22**	NFkB => NFkBn	5.4 min^{-1}	Import	-	(Werner et al., 2005)
**23**	IkBan => IkBa	0.012 min^{-1}	Export	-	(Werner et al., 2005)
**24**	IkBbn => IkBb	0.012 min^{-1}	Export	-	(Werner et al., 2005)
**25**	IkBen => IkBe	0.012 min^{-1}	Export	-	(Werner et al., 2005)
**79**	IkBdn => IkBd	0.012 min^{-1}	Export	-	(Shih et al., 2009)
**26**	NFkBn => NFkB	0.0048 min^{-1}	Export	-	(Werner et al., 2005)
**27**	IkBaNFkB => IkBaNFkBn	0.276 min^{-1}	Import	-	(Werner et al., 2005)
**28**	IkBbNFkB => IkBbNFkBn	0.0276 min^{-1}	Import	-	(Werner et al., 2005)
**29**	IkBeNFkB => IkBeNFkBn	0.138 min^{-1}	Import	-	(Werner et al., 2005)
**80**	IkBdNFkB => IkBdNFkBn	0.276 min^{-1}	Import	-	(Shih et al., 2009)
**30**	IkBaNFkBn => IkBaNFkB	0.828 min^{-1}	Export	-	(Werner et al., 2005)
**31**	IkBbNFkBn => IkBbNFkB	0.414 min^{-1}	Export	-	(Werner et al., 2005)
**32**	IkBeNFkBn => IkBeNFkB	0.414 min^{-1}	Export	-	(Werner et al., 2005)
**81**	IkBdNFkBn => IkBdNFkB	0.414 min^{-1}	Export	-	(Shih et al., 2009)
IκB Protein Degradation Reactions					
**33**	IkBa =>	0.12 min^{-1}	Prot. Deg.	Cytoplasm	(O'Dea et al., 2007)
**34**	IkBb =>	0.18 min^{-1}	Prot. Deg.	Cytoplasm	(O'Dea et al., 2007)
**35**	IkBe =>	0.18 min^{-1}	Prot. Deg.	Cytoplasm	(O'Dea et al., 2007)
**82**	IkBd =>	1.4E-3 min^{-1}	Prot. Deg.	Cytoplasm	(Shih et al., 2009)
**36**	IkBan =>	0.12 min^{-1}	Prot. Deg.	Nucleus	(O'Dea et al., 2007)
**37**	IkBbn =>	0.18 min^{-1}	Prot. Deg.	Nucleus	(O'Dea et al., 2007)
**38**	IkBen =>	0.18 min^{-1}	Prot. Deg.	Nucleus	(O'Dea et al., 2007)
**93**	IkBdn =>	1.4E-3 min^{-1}	Prot. Deg.	Nucleus	(Shih et al., 2009)
**39**	IkBaNFkB => NFkB	6E-5 min^{-1}	Prot. Deg.	Cytoplasm	(O'Dea et al., 2007)
**40**	IkBbNFkB => NFkB	6E-5 min^{-1}	Prot. Deg.	Cytoplasm	(O'Dea et al., 2007)
**41**	IkBeNFkB => NFkB	6E-5 min^{-1}	Prot. Deg.	Cytoplasm	(O'Dea et al., 2007)
**84**	IkBdNFkB => NFkB	6E-5 min^{-1}	Prot. Deg.	Cytoplasm	(Shih et al., 2009)
**42**	IkBaNFkBn => NFkBn	6E-5 min^{-1}	Prot. Deg.	Nucleus	(O'Dea et al., 2007)
**43**	IkBbNFkBn => NFkBn	6E-5 min^{-1}	Prot. Deg.	Nucleus	(O'Dea et al., 2007)
**44**	IkBeNFkBn => NFkBn	6E-5 min^{-1}	Prot. Deg.	Nucleus	(O'Dea et al., 2007)
**85**	IkBdNFkBn => NFkBn	6E-5 min^{-1}	Prot. Deg.	Nucleus	(Shih et al., 2009)
IκB:NFκB Association and Dissociation Reactions					
**45**	IkBa + NFkB => IkBaNFkB	30 $\mu M^{-1} min^{-1}$	Association	Cytoplasm	(Hoffmann et al., 2002)
**46**	IkBb + NFkB => IkBbNFkB	30 $\mu M^{-1} min^{-1}$	Association	Cytoplasm	(Hoffmann et al., 2002)
**47**	IkBe + NFkB => IkBeNFkB	30 $\mu M^{-1} min^{-1}$	Association	Cytoplasm	(Hoffmann et al., 2002)
**86**	IkBd + NFkB => IkBdNFkB	30 $\mu M^{-1} min^{-1}$	Association	Cytoplasm	(Shih et al., 2009)
**48**	IkBan + NFkBn => IkBaNFkBn	30 $\mu M^{-1} min^{-1}$	Association	Nucleus	(Hoffmann et al., 2002)
**49**	IkBbn + NFkBn => IkBbNFkBn	30 $\mu M^{-1} min^{-1}$	Association	Nucleus	(Hoffmann et al., 2002)
**50**	IkBen + NFkBn => IkBeNFkBn	30 $\mu M^{-1} min^{-1}$	Association	Nucleus	(Hoffmann et al., 2002)
**87**	IkBdn + NFkBn => IkBdNFkBn	30 $\mu M^{-1} min^{-1}$	Association	Nucleus	(Shih et al., 2009)
**51**	IkBaNFkB => IkBa + NFkB	6E-5 min^{-1}	Dissociation	Cytoplasm	(Hoffmann et al., 2002)
**52**	IkBbNFkB => IkBb + NFkB	6E-5 min^{-1}	Dissociation	Cytoplasm	(Hoffmann et al., 2002)
**53**	IkBeNFkB => IkBe + NFkB	6E-5 min^{-1}	Dissociation	Cytoplasm	(Hoffmann et al., 2002)
**88**	IkBdNFkB => IkBd + NFkB	6E-5 min^{-1}	Dissociation	Cytoplasm	(Shih et al., 2009)
**54**	IkBaNFkBn => IkBan + NFkBn	6E-5 min^{-1}	Dissociation	Nucleus	(Hoffmann et al., 2002)
**55**	IkBbNFkBn => IkBbn + NFkBn	6E-5 min^{-1}	Dissociation	Nucleus	(Hoffmann et al., 2002)
**56**	IkBeNFkBn => IkBen + NFkBn	6E-5 min^{-1}	Dissociation	Nucleus	(Hoffmann et al., 2002)
**89**	IkBdNFkBn => IkBdn + NFkBn	6E-5 min^{-1}	Dissociation	Nucleus	(Shih et al., 2009)
IKK-mediated IκB Degradation Reactions					
**57**	IkBa =>	0.36 min^{-1}	Prot. Deg.	Cytoplasm	(Mathes et al, 2008)
**58**	IkBb =>	0.12 min^{-1}	Prot. Deg.	Cytoplasm	(Mathes et al, 2008)
**59**	IkBe =>	0.18 min^{-1}	Prot. Deg.	Cytoplasm	(Mathes et al, 2008)
**90**	IkBdIKK1 => IKK1	1.2E-3 min^{-1}	Prot. Deg.	Cytoplasm	(Shih et al., 2009)
**60**	IkBaNFkB => NFkB	0.36 min^{-1}	Prot. Deg.	Cytoplasm	(Hoffmann et al., 2002)
**61**	IkBbNFkB => NFkB	0.12 min^{-1}	Prot. Deg.	Cytoplasm	(Hoffmann et al., 2002)
**62**	IkBeNFkB => NFkB	0.18 min^{-1}	Prot. Deg.	Cytoplasm	(Hoffmann et al., 2002)
**91**	IkBdIKK1NFkB => IKK1 + NFkB	0.18 min^{-1}	Prot. Deg.	Cytoplasm	(Shih et al., 2009)
A20 mRNA and Protein Synthesis and Degradation Reactions					
**63**	=> A20t (constitutive)	2 E-6 min^{-1}	RNA Synth.	-	Refer to #1.
**64**<br>**65**<br>**66**<br>**71**	=> A20t (induced by NFkBn)	0.4 $\mu M^{-2} min^{-1}$<br>Hill Coefficient: 3.0<br>Delay: 0 min<br>Shutdown: 120 min	RNA Synth.	-	- Refer to #1.<br>- Refer to #1.<br>- Refer to #1.<br>- A20 inducible transcription, as measured by RPA, appears to halt abruptly 2hrs into TNF stimulation.
**67**	A20t =>	0.035 min^{-1}	RNA Deg.	Cytoplasm	Refer to #1.
**68**<br>**69**	=> A20	0.25 min^{-1}<br>Delay Time: 30 min	Prot. Synth.	Cytoplasm	- Assumed to be equal to IkB translation rates.<br>- Delay was added to account for time between A20 mRNA expression as measured by RPA and A20 protein expression as measured by Western Blot.
**70**	A20 =>	0.0029 min^{-1}	Prot. Deg.	Cytoplasm	(Werner et al., 2005)

IKK Activation Module					
TNF-Independent Complex I Activity Reactions					
**2**	=> tnfrm	2 E-7 min^{-1}	Prot. Synth.	Cell Surface	Parameter value fit to recapitulate the measured steady-state amount of TNF receptor (Watanabe et al. 1988)
**3**	tnfrm =>	0.0058 min^{-1}	Prot. Deg.	Cell Surface	Measured in (Watanabe et al. 1988)
**4**	3 tnfrm => TNFR	1 E-5 μM^{-1} min^{-1}	Association	Cell Surface	Parameter value fit to account for minimal TNF receptor aggregation in the absence of ligand as observed in numerous published studies.
**5**	TNFR => 3 tnfrm	0.1 min^{-1}	Dissociation	Cell Surface	Refer to #4.
**6**	TNFR => (internalization)	0.0017 min^{-1}	Prot. Deg.	Cell Surface	Based upon results published in (Watanabe et al., 1988) showing that the temporal profile of TNF receptor following TNF stimulation.
**7**	TNFR + TTR => C1_off	100 μM^{-1} min^{-1}	Association	Cell Surface	Recruitment of TRAF2, TRADD, and RIP adaptors (TTR) to TNFR is required (but not sufficient) for signaling by the TNFR-containing signaling complex (C1). Little biophysical data is available for this reaction; recruitment appears to be simultaneous (Schneider-Brachert et al., 2004). The parameter value represents a compound mechanistic rate constant. It was fit to enable quick activation of downstream IKK activity within the first minutes of stimulation and repression upon removal of TNF ligand in pulse stimulations.
**8**	C1_off => TNFR + TTR	0.75 min^{-1}	Dissociation	Cell Surface	Refer to #7.
**9**	C1_off => C1	30 min^{-1}	Activation	Cell Surface	The molecular complex containing TNFR, TRAF2, TRADD and RIP undergoes an activation step that involves K63-ubiqutination of RIP. Little biophysical data is available for this step, but parameter fitting was constrained by the fast activation profile of IKK.
**10**	C1 => C1_off	2.0 min^{-1}	Deactivation	Cell Surface	Refer to #9.
**11**	C1 => C1_off (A20 mediated)	1000 μM^{-1} min^{-1}	Deactivation	Cell Surface	A20 is known to repress the activity of Complex I. It is a protease of K63-linked ubiquitin chains that deubiquitinates RIP (Wertz et al., 2004). Little biophysical data is available for this step, but parameter fitting was constrained by the IKK activity profiles measured in wild type and $a20^{-/-}$ cells.
**12**	C1 => TNFR + TTR	0.75 min^{-1}	Dissociation	Cell Surface	Assumed to be equal to #8.
**13**	C1_off => (internalization)	0.0017 min^{-1}	Prot. Deg.	Cell Surface	Assumed to be equal to #6.
**14**	C1 => (internalization)	0.0017 min^{-1}	Prot. deg.	Cell Surface	Assumed to be equal to #6.
TNF-Dependent Complex I Activity Reactions					
**1**	tnf =>	0.0154 min^{-1}	Prot. deg.	Extracellular	The half-life of recombinant TNF ligand in cell culture medium was measured by its manufacturer, Roche Diagnostics, to be 45-minutes.
**15**	tnf + 3 tnfrm => TNFRtnf	1100 μM^{-1} min^{-1}	Association	Cell Surface	Measured in (Grell et al., 1998).
**16**	tnf + TNFR => TNFRtnf	1100 μM^{-1} min^{-1}	Association	Cell Surface	Assumed to be equal to #15.
**17**	TNFRtnf => TNFR + tnf	0.021 min^{-1}	Dissociation	Cell Surface	Measured in (Grell et al., 1998).
**18**	TNFRtnf => (internalization				Assumed to be equal to #6.
**19**	TNFRtnf + TTR => C1tnf_off	100 μM^{-1} min^{-1}	Association	Cell Surface	Assumed to be equal to #7.<br>TNF binding to the extra-cellular domain of TNFR monomers speeds up trimerization and stabilizes the trimer, but recruitment of the TTR complex to trimerized TNF receptor are assumed to proceed with the same kinetics regardless of the presence of TNF ligand.
**20**	C1tnf_off => TNFRtnf + TTR	0.75 min^{-1}	Dissociation	Cell Surface	Refer to #19. Assumed to be equal to #8.
**21**	C1tnf_off => C1tnf	30 min^{-1}	Activation	Cell Surface	Refer to #19. Assumed to be equal to #9.
**22**	C1tnf => C1tnf_off	2.0 min^{-1}	Deactivation	Cell Surface	Refer to #19. Assumed to be equal to #10.
**23**	C1tnf => C1tnf_off (A20 mediated)	1000 μM^{-1} min^{-1}	Deactivation	Cell Surface	Refer to #19. Assumed to be equal to #11.
**24**	C1tnf => TNFRtnf + TTR	0.75 min^{-1}	Dissociation	Cell Surface	Refer to #19. Assumed to be equal to #8.
**25**	C1tnf_off => (internalization)	0.0017 min^{-1}	Prot. deg.	Cell Surface	Refer to #19. Assumed to be equal to #6.
**26**	C1tnf => (internalization)	0.0017 min^{-1}	Prot. deg.	Cell Surface	Refer to #19. Assumed to be equal to #6.
**27**	C1tnf_off => C1_off + tnf	0.021 min^{-1}	Dissociation	Cell Surface	Assumed to be equal to #17.
**28**	C1_off + tnf=> C1tnf_off	1100 μM^{-1} min^{-1}	Association	Cell Surface	Assumed to be equal to #15.
**29**	C1tnf => C1 + tnf	0.021 min^{-1}	Dissociation	Cell Surface	Assumed to be equal to #17.
**30**	C1 + tnf => C1tnf	1100 μM^{-1} min^{-1}	Association	Cell Surface	Assumed to be equal to #15.

IKKK (TAB1/2-TAK1 complex) Activity Reactions					
**31**	IKKK_off => IKKK (constitutive)	5 E-7 min^{-1}	Activation	Cytoplasm	Parameter value fit to account for low IKK activity in the absence of ligand as measured by IKK Kinase Assay (O'Dea et al., 2007).
**32**	IKKK_off => IKKK (C1 mediated)	500 μM^{-1} min^{-1}	Activation	Cytoplasm	Refer to #7.
**33**	IKKK_off => IKKK (C1tnf mediated)	500 μM^{-1} min^{-1}	Activation	Cytoplasm	Refer to #19. Assumed to be equal to #32.
**34**	IKKK => IKKK_off (constitutive)	0.25 min^{-1}	Deactivation	Cytoplasm	The constitutive inactivation rate of this complex was fit to ensure low basal activity and efficient repression following TNF pulse stimulation.
IKK Activity Reactions					
**35**	IKK_off => IKK	5 E-5 min^{-1}	Activation	Cytoplasm	Refer to #31
**36**	IKK_off => IKK (IKKK mediated)	520 μM^{-1} min^{-1}	Activation	Cytoplasm	Refer to #7.
**37**	IKK => IKK_off	0.02 min^{-1}	Deactivation	Cytoplasm	Refer to #34.
**38**	IKK => IKK_i (self-inactivation)	0.15 min^{-1}	Deactivation	Cytoplasm	IKK is thought to down-regulate its own activity via auto-phosphorylation of C-terminal serine residues (Delhase et al., 1999). This mechanism was not shown to cause IKK protein degradation and is distinct from inactivating IKK via constitutive phosphatase activity (Refer to #94). The parameter value was fit to temporal profiles of IKK activity in response to TNF stimulation (Werner et al., 2005).
**39**	IKK_i => IKK_off	0.02 min^{-1}	Deactivation	Cytoplasm	C-terminally phosphorylated IKK is assumed to be subject to constitutive phosphatase activity. Refer to #38.

**Necroptosis module**					
Necroptosis Activity Reactions					
**92**	C1_off + C1 --> RIP1	6 min^{-1}	Activation	Cytoplasm	Fitted to data of RIP1 protein
**93**	A20 --| RIP3	6 min^{-1}	Inhibition	Cytoplasm	Fitted to data of A20 protein and RIP3 protein
**94**	RIP1 =>	0.6 μM^{-1} min^{-1}	Degradation	Cytoplasm	Fitted to data of RIP1 protein
**95**	RIP1 --> RIP3	6 min^{-1}	Activation	Cytoplasm	Fitted to data of RIP1 protein and RIP3 protein
**96**	RIP3 =>	6 μM^{-1} min^{-1}	Degradation	Cytoplasm	Fitted to data of RIP3 protein
**97**	RIP3 --> pMLKL	300 min^{-1}	Activation	Cytoplasm	Fitted to data of RIP3 protein and pMLKL protein
**98**	pMLKL =>	60 μM^{-1} min^{-1}	Degradation	Cytoplasm	Fitted to data of pMLKL protein

Note for Appendix Table 6: There are 98 equations in the model. The NFκB module and IKK activation module are modeled by chemical reactions by the law of mass action. The necroptosis module is coarse-grained as activation and inhibition regulatory network, which are modeled by using Hill functions.

Appendix Table 7: Perturbed parameters for NFκB module

# in NFκB model	Parameter	Perturbed ratio	Adjustment on Parameter Value
**1**	TNF decay rate	0.1	Fitted to death time distribution
**63**	A20 basal transcription	0.6	Fitted to data of A20 mRNA for L929 cells
**64**	A20 induced transcription	0.15	Fitted to data of A20 mRNA for L929 cells
**68**	A20 translation	20	Fitted to data of A20 protein for L929 cells
**2**	IκBβ basal transcription	0.85	Fitted to data of IκBβ mRNA for L929 cells
**5**	IκBβ induced transcription	20	Fitted to data of IκBβ mRNA for L929 cells
**72**	IκBδ basal transcription	0.3	Fitted to data of IκBδ mRNA for L929 cells
**73**	IκBδ induced transcription	4	Fitted to data of IκBδ mRNA for L929 cells

Appendix Table 8: Parameters for necroptosis module

Reaction #	Parameter	Parameter Value	Adjustment on Parameter Value
**92**	Synthesis rate k_{s1}	distributed	
**95**	Synthesis rate k_{s2}	6 min^{-1}	Fitted to data of RIP3 protein
**97**	Synthesis rate k_{s3}	300 min^{-1}	Fitted to data of RIP3 protein and pMLKL protein
**94**	Degradation rate k_{d1}	0.6 μM^{-1} min^{-1}	Fitted to data of RIP3 protein
**96**	Degradation rate k_{d2}	6 μM^{-1} min^{-1}	Fitted to data of RIP3 protein
**98**	Degradation rate k_{d3}	60 μM^{-1} min^{-1}	Fitted to data of pMLKL protein
**92**	Km_1	1 μM	Fitted to data of RIP3 protein
**95**	Km_2	0.15 μM	Fitted to data of RIP3 protein
**93**	Km_3	0.5 μM	Fitted to data of A20 protein and RIP3 protein
**97**	Km_4	0.3 μM	Fitted to data of RIP3 protein and pMLKL protein
**92**	Hill coefficient n_1	3	Fitted to data of RIP3 protein
**95**	Hill coefficient n_2	3	Fitted to data of RIP3 protein
**93**	Hill coefficient n_3	5	Fitted to data of A20 protein and RIP3 protein
**97**	Hill coefficient n_4	3	Fitted to data of RIP3 protein and pMLKL protein

Appendix Table 9: Distributed parameters for necroptosis model

Reaction #	Parameter	Parameter distribution
**92**	Synthesis rate k_{s1} from RIP1 to RIP3	lognrnd(0,0.5)*0.7, where lognrnd(a,b) is log normal distribution with mean a and variable b.
**64**	A20 induced transcription	0.0053*normal(3,30)+ 0.0036* normal(600,135) , where normal(a,b) is a normal distribution with mean a and variable b.

References

Alon, U. (2007). Network motifs: theory and experimental approaches. Nature Reviews Genetics *8*, 450–461.

Bintu, L., Buchler, N.E., Garcia, H.G., Gerland, U., Hwa, T., Kondev, J., and Phillips, R. (2005). Transcriptional regulation by the numbers: models. Current Opinion in Genetics & Development *15*, 116–124.

Friedman, N., Cai, L., and Xie, X.S. (2006). Linking Stochastic Dynamics to Population Distribution: An Analytical Framework of Gene Expression. Phys. Rev. Lett. *97*, 168302.

Goldbeter, A., and Koshland, D.E. (1981). An amplified sensitivity arising from covalent modification in biological systems. PNAS *78*, 6840–6844.

Gong, Y.-N., Guy, C., Olauson, H., Becker, J. U., Yang, M., Fitzgerald, P., Linkermann, A., Green, D.R. (2017). ESCRT-III acts downstream of MLKL to regulate necroptotic cell death and its consequences. Cell *169(2):* 286-300.e16.

Hartigan, J.A., and Hartigan, P.M. (1985). The Dip Test of Unimodality. Ann. Statist. *13*, 70–84.

Huang, C.Y., and Ferrell, J.E. (1996). Ultrasensitivity in the mitogen-activated protein kinase cascade. PNAS *93*, 10078–10083.

Kærn, M., Blake, W.J., and Collins, J.J. (2003). The Engineering of Gene Regulatory Networks. Annual Review of Biomedical Engineering *5*, 179–206.

Kapuy, O., Barik, D., Domingo Sananes, M.R., Tyson, J.J., and Novák, B. (2009). Bistability by multiple phosphorylation of regulatory proteins. Progress in Biophysics and Molecular Biology *100*, 47–56.

Krishna, S., Jensen, M.H., and Sneppen, K. (2006). Minimal model of spiky oscillations in NF-κB signaling. PNAS *103*, 10840–10845.

Ma, W., Trusina, A., El-Samad, H., Lim, W.A., and Tang, C. (2009). Defining Network Topologies that Can Achieve Biochemical Adaptation. Cell *138*, 760–773.

MacArthur, B.D., Ma'ayan, A., and Lemischka, I.R. (2009). Systems biology of stem cell fate and cellular reprogramming. Nature Reviews Molecular Cell Biology *10*, 672–681.

Roux, J., Hafner, M., Bandara, S., Sims, J.J., Hudson, H., Chai, D., Sorger, P.K. (2015). Fractional killing arises from cell-to-cell variability in overcoming a caspase activity threshold. Molecular Systems Biology *11(5)*: 803.

Samson, A.L., Zhang, Y., Geoghegan, N.D., Gavin, X.J., Davies, K.A., Mlodzianoski, M.J., Whitehead, L.W., Frank, D., Garnish, S.E., Fitzgibbon, C., Hempel, A., Young, S.N., Jacobsen, A.V., Cawthorne, W., Petrie, E.J., Faux, M.C., Shield-Artin, K., Lalaoui, N., Hildebrand, J.M., Silke, J., Rogers, K.L., Lessene, G., Hawkins, E.D., Murphy, J.M. (2020). MLKL trafficking and accumulation at the plasma membrane control the kinetics and threshold for necroptosis. Nature Communications *11(1):*3151.

Shalek, A.K., Satija, R., Adiconis, X., Gertner, R.S., Gaublomme, J.T., Raychowdhury, R., Schwartz, S., Yosef, N., Malboeuf, C., Lu, D., et al. (2013). Single-cell transcriptomics reveals bimodality in expression and splicing in immune cells. Nature *498*, 236–240.

Shih, V.F.-S., Kearns, J.D., Basak, S., Savinova, O.V., Ghosh, G., and Hoffmann, A. (2009). Kinetic control of negative feedback regulators of NF-κB/RelA determines their pathogen- and cytokine-receptor signaling specificity. PNAS *106*, 9619–9624.

Shu, J., Wu, C., Wu, Y., Li, Z., Shao, S., Zhao, W., Tang, X., Yang, H., Shen, L., Zuo, X., et al. (2013). Induction of Pluripotency in Mouse Somatic Cells with Lineage Specifiers. Cell *153*, 963–975.

Spencer, S.L., Gaudet, S., Albeck, J.G., Burke, J.M., and Sorger, P.K. (2009). Non-genetic origins of cell-to-cell variability in TRAIL-induced apoptosis. Nature *459*, 428–432.

Tang, Y., Yuan, R., Wang, G., Zhu, X., and Ao, P. (2017). Potential landscape of high dimensional nonlinear stochastic dynamics with large noise. Scientific Reports *7*, 1–11.

Tyson, J.J., and Novák, B. (2010). Functional Motifs in Biochemical Reaction Networks. Annual Review of Physical Chemistry *61*, 219–240.

Tyson, J.J., Chen, K.C., and Novak, B. (2003). Sniffers, buzzers, toggles and blinkers: dynamics of regulatory and signaling pathways in the cell. Current Opinion in Cell Biology *15*, 221–231.

Werner, S.L., Kearns, J.D., Zadorozhnaya, V., Lynch, C., O'Dea, E., Boldin, M.P., Ma, A., Baltimore, D., and Hoffmann, A. (2008). Encoding NF-κB temporal control in response to TNF: distinct roles for the negative regulators IκBα and A20. Genes Dev. *22*, 2093–2101.
