# Supplementary material for: An incoherent feedforward loop interprets NFκB/RelA dynamics to determine TNF‐induced necroptosis decisions
Source: Mol Syst Biol. 2020 Dec 14;16(12):e9677. doi: 10.15252/msb.20209677 (PMC7734648; doi:10.15252/msb.20209677)
Supplement: Supplementary file 2 — Expanded View Figures PDF [file MSB-16-e9677-s002.pdf]

Expanded View Figures

Figure EV1. NECtrack measures pro-inflammatory necroptotic cell death.

A Immune threats or tissue injury generate pathogen-associated molecular patterns (PAMPs) or damage-associated molecular patterns (DAMPs) to initiate inflammatory response. Tissue-resident macrophages respond with combined activation of NFκB and MAPK p38 pathways, and produce key inflammatory cytokines including IL6, IL1, and TNF. Stromal cells including fibroblasts perceive those cytokines and participate in response to inflammation. TNF-induced NFκB signaling drives release of chemokines (CCLs), matrix metalloproteinases (MMPs), and death ligands (FASL) to coordinate and resolve inflammatory cell infiltration. Alternatively, TNF may induce necroptotic cell death via RIP signaling, which generates more DAMPs to amplify inflammatory response. Whether TNF-induced NFκB cross-regulates necroptosis is unclear.

B Proliferative index of L929 wild-type (wt) cells treated with TNF obtained from live-cell microscopy (mean of three independent experiments ± standard deviation).

C Fractional cellular survival after 24 h TNF in the presence or absence of fluorescent DNA staining (Hoechst and PI) quantified by crystal violet assay (mean of three independent experiments ± standard deviation; two-tailed Student's *t*-test revealed no statistically significant differences, n.s., $P > 0.05$).

D Fractional survival of TNF-treated L929 wt cells obtained via two independent methods (mean of three independent experiments ± standard deviation).

E L929 wt cells were treated with 10 ng/ml TNF for 16 h. Cell lysates were diluted in lysis buffer (LB) and analyzed for pMLKL via immunoblot using the primary antibody ab196436 (Abcam). Relative quantification of the signal demonstrates an approximate linear relationship between pMLKL concentrations and detected signal.

F Immunoblot for pMLKL in L929 wt cells (representative data of three independent experiments).

G Two-phased death rates in a clonal L929 wt cell population (mean of three independent experiments ± standard deviation).

H TNF-induced NFκB activity in L929 wt cells measured via EMSA (representative data of three independent experiments).

I Antibody against RelA, but not RelB induces shift of bands, revealing they largely consist of p50:RelA (representative data of three independent experiments).

J Normalized RelA activity measured via EMSA and death rates in time course of TNF treatment (means of three independent experiments ± standard deviation).

K Immunoblot for RelA in L929 wt and CRISPR/Cas9 RelA-knockout (RelA KO) cell lines including clonal RelA KO population (asterisk indicates unspecific band present in all lanes).

L Fractional survival after 24 h of TNF treatment (mean of three independent experiments ± standard deviation; two-tailed Student's *t*-test **$P < 0.01$).

M Immunoblot for pMLKL (dash indicates empty lane; representative data of three independent experiments).

N Representative images illustrating morphological characteristics of apoptosis vs. necrosis in RelA KO cells. Apoptotic cell (t1, left) shows cytoplasmic blebbing (t2) and delayed PI positivity of nucleus (t3) indicative of secondary necrosis. Necrotic cell (right, t1) shows cytoplasmic swelling and rapid PI positivity (t2). Differential interference contrast, DIC.

O Quantification of apoptotic vs. necrotic death in L929 wt and RelA KO cells manually curated from live-cell microscopy experiments using morphological criteria shown in (N) (200 cells analyzed per experiment; mean of three independent experiments ± standard deviation).

Source data are available online for this figure.

 © 2020 The Authors

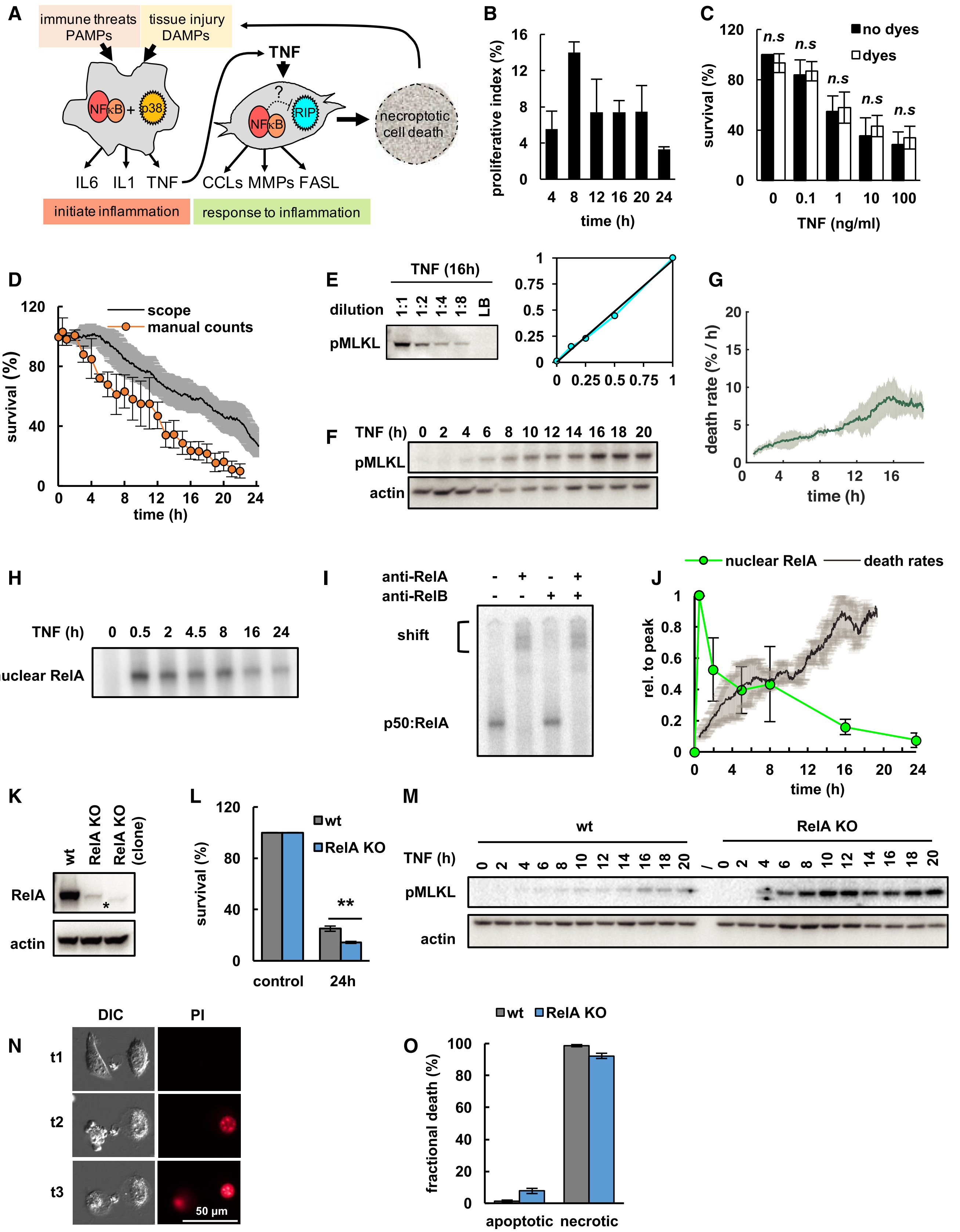
A
immune threats PAMPs
tissue injury DAMPs
TNF
NFκB + p38
NFκB
RIP
necroptotic cell death
IL6 IL1 TNF
CCLs MMPs FASL
initiate inflammation
response to inflammation
B
proliferative index (%)
time (h)
C
survival (%)
no dyes
dyes
n.s
TNF (ng/ml)
D
scope
manual counts
survival (%)
time (h)
E
TNF (16h)
dilution
1:1 1:2 1:4 1:8 LB
pMLKL
F
TNF (h)
pMLKL
actin
G
death rate (% / h)
time (h)
H
TNF (h)
nuclear RelA
I
anti-RelA
anti-RelB
shift
p50:RelA
J
nuclear RelA
death rates
rel. to peak
time (h)
K
wt
RelA KO
RelA KO (clone)
RelA
actin
L
wt
RelA KO
survival (%)
control
24h
M
wt
RelA KO
TNF (h)
pMLKL
actin
N
DIC
PI
t1
t2
t3
50 µm
O
wt
RelA KO
fractional death (%)
apoptotic
necrotic

Figure EV1.

Figure EV2. RelA mediates protection from necroptosis through TNF-inducible A20.

A, B Fractional survival quantified by crystal violet assay after treatment with TNF and (A) ROS inhibitor (ROSi), (B) JNK inhibitor (JNKi), and/or ZVAD (mean of three independent experiments ± standard deviation; two-tailed Student's t-test *$P < 0.05$, **$P < 0.01$, ***$P < 0.001$, or no statistically significant difference, n.s., $P > 0.05$).

C TNF-induced death rates in L929 wild-type cells with and without ZVAD pre-treatment obtained via live-cell microscopy (mean of three independent experiments ± standard deviation).

D TNF-induced mRNA expression measured via qRT–PCR (mean of three independent experiments ± standard deviation; two-tailed Student's t-test revealed no statistically significant results, $P > 0.05$).

E Immunoblot for FLIP-L (full) and its cleavage product (p43; representative data of three independent experiments).

F Relative mRNA expression of A20 or cIAP2 measured via qRT–PCR, and normalized RelA activity measured via EMSA in wt cells treated with TNF (mean of three independent experiments ± standard deviation).

G Immunoblot for A20 protein (representative data of three independent experiments).

H Whole cell lysates depleted of RIPK3-containing protein complexes using anti-RIPK3 served as input for RIPK1 co-immunoprecipitation (IP) and subsequent immunoblot analysis. Flow through, FT. Isotype control, IgG rb.

I Representative microscopy images of smFISH experiments for the NFκB-responsive target genes A20 (red spots) and IκBα (green spots) in wt cells treated with TNF (contrast set to same limits for every time point).

J Histogram of volume normalized mRNA copy numbers measured by smFISH (two independent experiments; first independent experiment shown in Fig 2D).

K, L (K) Immunoblot for cIAP2 using commercially available Pan-cIAP antibody and cIAP1-knockout cell line as control. As cIAP2 protein is below detection limit, CRISPR/Cas9-mediated knockout was validated (L) via high-resolution melt (HRM) analysis.

M TNF-induced death rates obtained via live-cell microscopy (mean of three independent experiments ± standard deviation).

Source data are available online for this figure.

 © 2020 The Authors

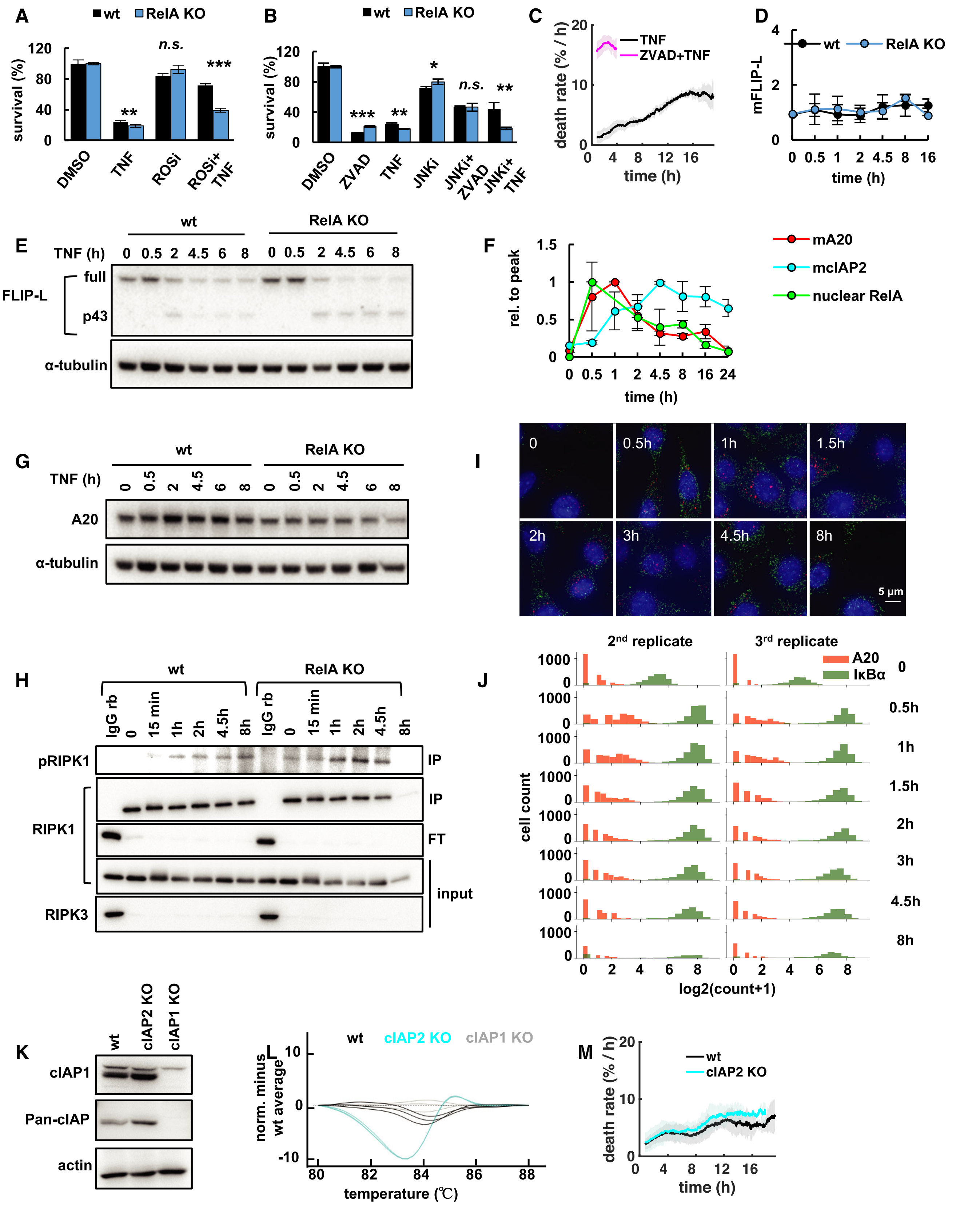
A
wt RelA KO
survival (%)
DMSO
TNF
ROSi
ROSi+ TNF
n.s.
B
wt RelA KO
survival (%)
DMSO
ZVAD
TNF
JNKi
JNKi+ ZVAD
JNKi+ TNF
C
death rate (% / h)
TNF
ZVAD+TNF
time (h)
D
wt RelA KO
mFLIP-L
time (h)
E
wt RelA KO
TNF (h)
FLIP-L
full
p43
α-tubulin
F
mA20
mcIAP2
nuclear RelA
rel. to peak
time (h)
G
wt RelA KO
TNF (h)
A20
α-tubulin
I
5 μm
H
wt RelA KO
IgG rb
15 min
pRIPK1
RIPK1
RIPK3
IP
FT
input
J
2nd replicate
3rd replicate
A20
IκBα
cell count
log2(count+1)
K
wt
cIAP2 KO
cIAP1 KO
cIAP1
Pan-cIAP
actin
L
norm. minus wt average
temperature (℃)
M
wt
cIAP2 KO
death rate (% / h)
time (h)

Figure EV2.

Figure EV3. Mathematical model quantitatively accounts for RelA-A20 controlled necroptosis kinetics.

A Detailed schematic diagram of model reaction network (for reactions and parameters see Tables in Appendix).

B–D Model simulations (smoothed line represents population average, and shaded area the 20th percentile around the median) and experimental measurements (data points, mean of three independent experiments ± standard deviation) of indicated species over the time course of TNF treatment in L929 wild-type cells. A.U., arbitrary units.

E Immunoblot for pMLKL and RIPK3 in detergent-soluble and -insoluble fractions of wild-type cells as a biochemical correlate of the active necrosome (representative data of three independent experiments).

F, G Simulations (smoothed line represents population average, and shaded area the 20th percentile around the median) and relative quantification of experimental measurements of necrosome activity in (E) (mean of three independent experiments ± standard deviation).

H One representative of three immunoblots of A20 in wild-type (wt), parental RelA-knockout (KO) cells (–) or RelA KO cells expressing A20 from a constitutive transgene (pBabe-A20, +). Relative quantification across three independent experiments (mean ± standard deviation; two-tailed Student's *t*-test *$P < 0.05$, or no statistically significant difference, n.s., $P > 0.05$).

I Probability of unimodal distributions of death times calculated by Hartigan's dip significance (mean of three independent experiments ± standard deviation; two-sample *t*-test revealed no statistical significance, n.s., $P > 0.05$).

J A20 immunoblot in wt or A20 KO cells (left). Simulated and measured distribution of death times in A20 KO cells (representative data of three independent experiments).

K Immunoblot of A20 in wt and A20 KO cells reconstituted with an NFκB-inducible transgene (fIL8-A20) and relative quantification (mean of three independent experiments ± standard deviation).

L Probability of unimodal distributions of death times calculated by Hartigan's dip significance in parental A20 KO cells (–) or A20 KO cells expressing fIL8-A20 (+). Mean of three independent experiments ± standard deviation; two-sample *t*-test **$P < 0.01$).

M 24-h fractional survival in response to varying concentrations of TNF (mean of three independent experiments ± standard deviation).

Source data are available online for this figure.

 © 2020 The Authors

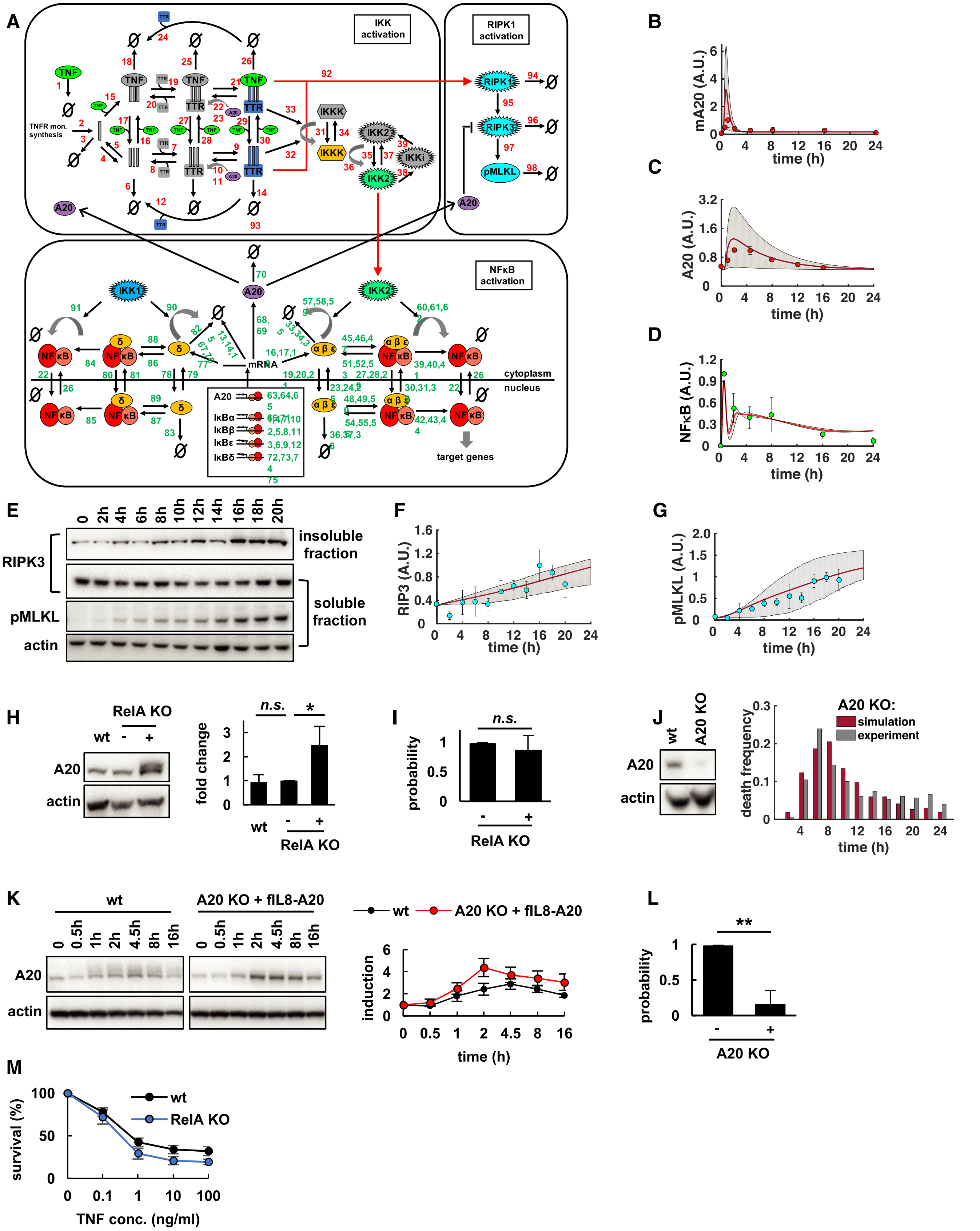

Figure EV3.

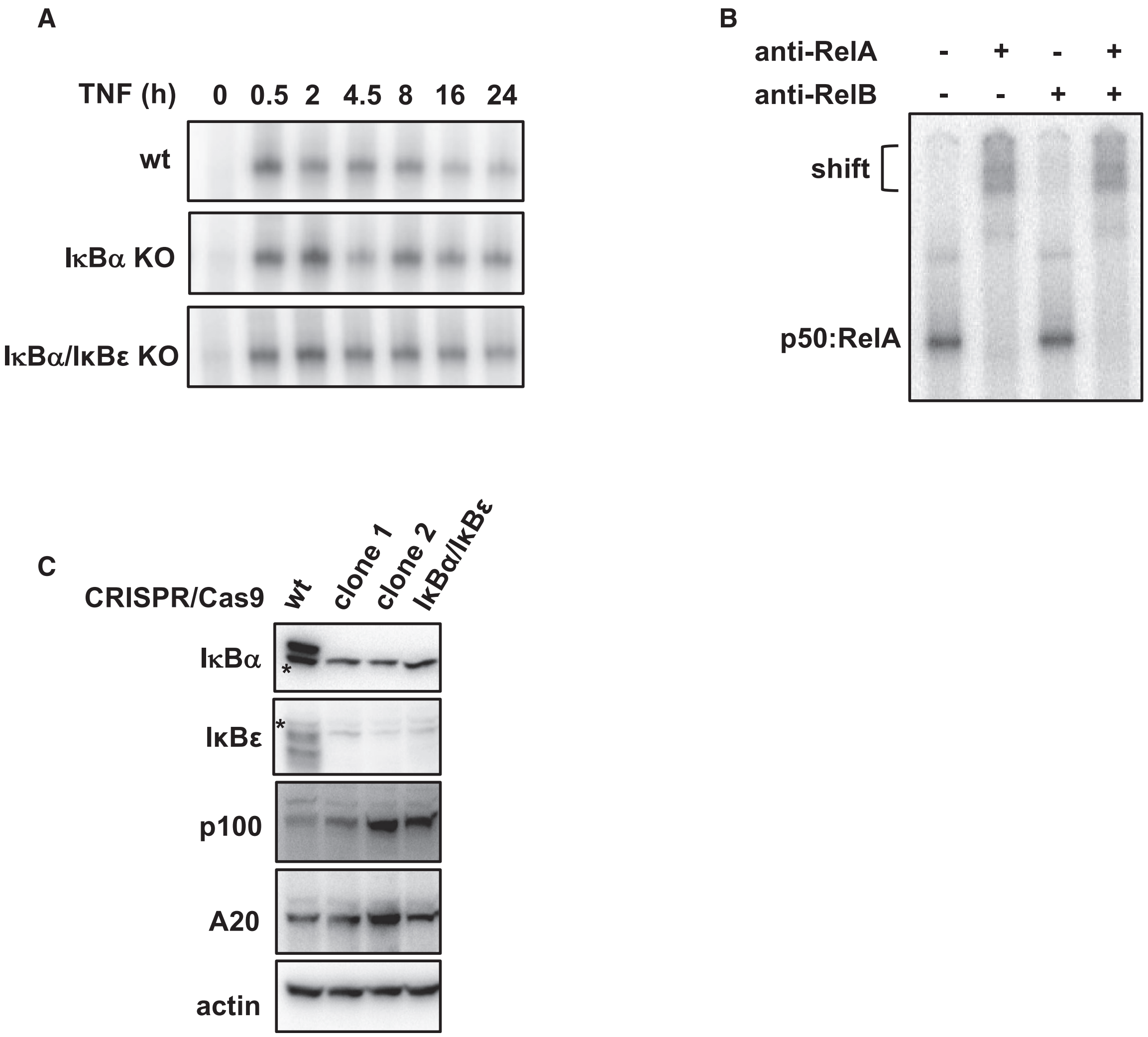

Figure EV4. Dysregulated NFκB dynamics in IκB-knockout cells.

A TNF-induced NFκB activity in indicated cell lines measured via EMSA (representative data of three independent experiments).
B Antibody for RelA, but not RelB induces shift of bands, revealing they largely consist of p50:RelA (representative data of three independent experiments).
C Immunoblot for indicated proteins; clonal population 1 (clone 1) was selected for maintaining basal expression of p100 and A20 despite of IκBα/IκBε-knockout. Asterisks depict unspecific bands.

Source data are available online for this figure.

Figure EV5. Pathophysiological consequences of the NFκB-A20-RIPK3 circuit.

A In normal tissues, the NFκB-A20-RIPK3 circuit protects the majority of fibroblasts from death induced by transient signals of TNF to participate in the coordination and resolution of acute inflammation (left). In conditions of prolonged inflammation, sustained TNF signals overcome the protective NFκB-A20-RIPK3 circuit, leading to massive necroptosis, the release of DAMPs, and overwhelming inflammation, which may contribute to inflammatory diseases and tissue damage (right).
B In tumors with normal NFκB activity, sustained TNF signals may induce sufficient levels of necroptotic cell death to establish immunogenicity and an effective anti-tumor response (left). In contrast, in tumors with dysregulated NFκB activity, prolonged expression of A20 may contribute to low immunogenicity and avoidance of immune destruction (right).

© 2020 The Authors

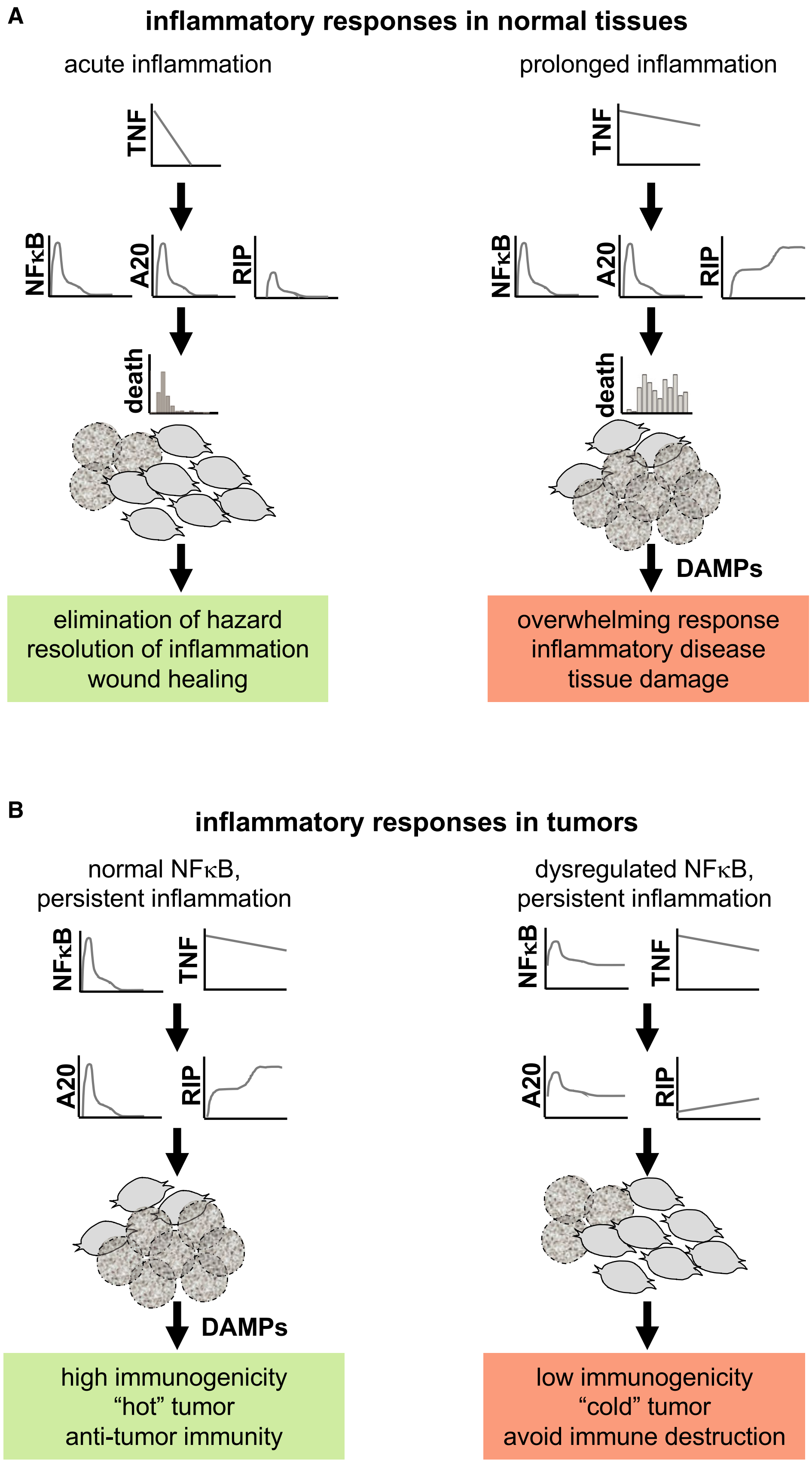
A
inflammatory responses in normal tissues
acute inflammation
prolonged inflammation
TNF
NFκB
A20
RIP
death
DAMPs
elimination of hazard
resolution of inflammation
wound healing
overwhelming response
inflammatory disease
tissue damage
B
inflammatory responses in tumors
normal NFκB,
persistent inflammation
dysregulated NFκB,
persistent inflammation
high immunogenicity
"hot" tumor
anti-tumor immunity
low immunogenicity
"cold" tumor
avoid immune destruction

Figure EV5.
